# Development of Rice Variety With Durable and Broad-Spectrum Resistance to Blast Disease Through Marker-Assisted Introduction of *Pigm* Gene

**DOI:** 10.3389/fpls.2022.937767

**Published:** 2022-07-22

**Authors:** Zhiming Feng, Mingyou Li, Zhiwen Xu, Peng Gao, Yunyu Wu, Keting Wu, Jianhua Zhao, Xiaoqiu Wang, Jianan Wang, Mengchen Li, Keming Hu, Hongqi Chen, Yiwen Deng, Aihong Li, Zongxiang Chen, Shimin Zuo

**Affiliations:** ^1^Key Laboratory of Plant Functional Genomics of the Ministry of Education/Jiangsu Key Laboratory of Crop Genomics and Molecular Breeding, Agricultural College of Yangzhou University, Yangzhou, China; ^2^Co-innovation Center for Modern Production Technology of Grain Crops of Jiangsu Province/Key Laboratory of Crop Genetics and Physiology of Jiangsu Province, Yangzhou University, Yangzhou, China; ^3^Institute of Agricultural Sciences for Lixiahe Region in Jiangsu, Yangzhou, China; ^4^State Key Laboratory of Rice Biology, China National Rice Research Institute, Hangzhou, China; ^5^National Key Laboratory of Plant Molecular Genetics, CAS Center for Excellence in Molecular Plant Sciences, Institute of Plant Physiology and Ecology, Chinese Academy of Sciences, Shanghai, China; ^6^Joint International Research Laboratory of Agriculture and Agri-Product Safety, The Ministry of Education of China, Institutes of Agricultural Science and Technology Development, Yangzhou University, Yangzhou, China

**Keywords:** rice, blast resistance, *Pigm* gene, marker-assisted selection, *geng* rice in Jiangsu

## Abstract

Rice blast, caused by *Magnaporthe oryzae* (*M. oryzae*), is one of the most destructive diseases threatening rice production worldwide. Development of resistant cultivars using broad-spectrum resistance (*R*) genes with high breeding value is the most effective and economical approach to control this disease. In this study, the breeding potential of *Pigm* gene in *geng*/*japonica* rice breeding practice in Jiangsu province was comprehensively evaluated. Through backcross and marker-assisted selection (MAS), *Pigm* was introduced into two *geng* rice cultivars (Wuyungeng 32/WYG32 and Huageng 8/HG8). In each genetic background, five advanced backcross lines with *Pigm* (ABLs) and the same genotypes as the respective recurrent parent in the other 13 known *R* gene loci were developed. Compared with the corresponding recurrent parent, all these ABLs exhibited stronger resistance in seedling inoculation assay using 184 isolates collected from rice growing regions of the lower region of the Yangtze River. With respect to panicle blast resistance, all ABLs reached a high resistance level to blast disease in tests conducted in three consecutive years with the inoculation of seven mixed conidial suspensions collected from different regions of Jiangsu province. In natural field nursery assays, the ABLs showed significantly higher resistance than the recurrent parents. No common change on importantly morphological traits and yield-associated components was found among the ABLs, demonstrating the introduction of *Pigm* had no tightly linked undesirable effect on rice economically important traits and its associated grain weight reduction effect could be probably offset by others grain weight genes or at least in the background of the aforementioned two varieties. Notably, one rice line with *Pigm*, designated as Yangnonggeng 3091, had been authorized as a new variety in Jiangsu province in 2021, showing excellent performance on both grain yield and quality, as well as the blast resistance. Together, these results suggest that the *Pigm* gene has a high breeding value in developing rice varieties with durable and broad-spectrum resistance to blast disease.

## Introduction

Rice blast, caused by the fungus *Magnaporthe oryzae* (*M. oryzae*), is one of the most devastating diseases of rice worldwide (Dean et al., 2012). The yield loss caused by rice blast was 10–30% in epidemic years and reached 40–50% or even higher in serious cases (Skamnioti and Gurr, 2009; Li et al., 2019). For example, it brought approximately 566 thousand tons of yield loss in 2015 alone in China (Liu et al., 2016a). Developing resistant cultivars by marker-assisted selection (MAS) of resistance genes has been proven to be the most effective, economical and eco-friendly strategy to control blast disease (Xiao et al., 2020).

To date, more than 350 quantitative resistance loci (*QRLs*) and 100 qualitative resistance (*R*) genes conferring resistance to *M. oryzae* have been identified, of which at least 32 *R* genes and five *QRL* genes have been cloned (Li et al., 2017; Yin et al., 2021). Comparatively, *QRL* genes, like *Pi21* and *bsr-d1*, generally have broad-spectrum and durable resistance because they mostly do not belong to ‘gene-for-gene’ resistance and do not exert strong selection pressure on pathogens. Through genome editing, Tao et al. (2021) and Zhou et al. (2021a) obtained some lines that contained either of the two genes, *Pi21* and *bsr-d1*, or both. After inoculation, they found that the lines containing both *QRL* genes showed apparently stronger resistance than the lines possessing one of them, implying the pyramiding of *QRL* genes is a feasible approach to develop a broad-spectrum resistance variety. However, due to relatively small effects of *QRL* genes on resistance, especially on panicle resistance, as well as limited number of *QRL* genes isolated so far, deployment of *R* genes with broad-spectrum resistance is of particular importance in current rice breeding projects. To date, only a few *R* genes were considered conferring broad-spectrum resistance, such as *Piz*, *Piz-t*, *Pi1*, *Pi2*, *Pi9*, *Pi33*, *Pi54*, *Pigm*, and *Pi40* (Li et al., 2020; Xiao et al., 2020). Among them, *Pigm*, isolated from a Chinese local variety Gumei 4 with broad-spectrum and durable resistance to blast, exhibited resistance to 50 isolates of *M. oryzae* from all over the world (Deng et al., 2006, 2017). It is particularly interesting since it contains a pair of antagonistic genes, *PigmR* and *PigmS*. *PigmR* gives rice broad-spectrum resistance, but it reduces rice 1000-seed weight and affects grain yield. *PigmS*, in contrast, offsets the adverse effect of *PigmR* on yield by improving the seed setting. More importantly, PigmS inhibits PigmR-mediated broad-spectrum disease resistance by competing with PigmR to form a heterodimer, which alleviates the selection pressure of the pathogenic evolution of *M. oryzae* races and so renders *PigmR* durable resistance (Deng et al., 2017), implying its importance in rice breeding program.

Leaf blast and panicle blast are two major types of rice blast diseases, and panicle blast is an important cause for yield loss because it occurs on the panicle neck, directly restricting grain filling (Titone et al., 2015). Resistance to panicle blast and leaf blast at the seedling stage is often inconsistent, which means that the resistance of some *R* genes is not effective across the development stages (Puri et al., 2009; Ishihara et al., 2014; Liu et al., 2016b). Therefore, it is particularly important to employ broad-spectrum *R* genes independent of the development stage in rice molecular breeding. However, most studies have focused on seedling blast resistance, rather than panicle blast resistance, because the panicle inoculation generally needs a large space and is time-consuming and easily affected by the environment compared to spraying inoculation at the seedling stage (Hao et al., 2011; Xiao et al., 2020). Most of the broad-spectrum *R* genes mentioned before have not been evaluated for panicle blast resistance. Recently, a set of near-isogenic lines carrying six resistance alleles (*Piz*, *Piz-t*, *Pi2*, *Pi9*, *Pi40*, and *Pigm*) at the *Piz* locus, respectively, under the background of *xian/indica* rice variety Yangdao 6 and *geng/japonica* rice line 07GY31, were developed by Wu et al. (2016, 2017). After inoculation with *M. oryzae* isolates from different ecological regions of China, they found that *Pigm* showed more broad-spectrum resistance to both seedling blast and panicle blast than the other *R* genes (Wu et al., 2016, 2017).

*Geng* rice is an important food source in China, and more than 85% of rice cultivation areas grow *geng* rice in Jiangsu province (Zhang et al., 2011). Recently, the occurrence of rice blast, especially for panicle blast, has presented an increasingly serious threat on rice production in Jiangsu province. For instance, around 70% of rice fields were destroyed by panicle blast in 2014, resulting in huge yield losses (Wei and Song, 2014; He et al., 2015). Studies have shown that some *R* genes, like *Pib*, *Pita*, *Pish*, *Pik-h*, *Piz*, and *Pi54*, have been more widely used in *geng* rice varieties authorized in Jiangsu province (Fan et al., 2014; Wu et al., 2015; Wang et al., 2020), while with the genetic variation of *M. oryzae* strains, these *R* genes are gradually losing their resistance (Zhu et al., 2018; Wang et al., 2020). For the *R* gene *Pigm*, however, to the best of our knowledge, almost no *geng* varieties currently released in Jiangsu province contain this gene. Recently, Zeng et al. (2018) introduced *Pigm* into three different *geng* rice varieties through MAS and found that the BC_4_F_1_ plants with heterozygous *Pigm* alleles showed strong panicle blast resistance against *M. oryzae* races from southern China. In addition, Wang et al. (2019) and Chen et al. (2020) employed two different *geng* rice varieties from Jiangsu province to cross or backcross with Gumei 4 and found that the plants carrying *Pigm* gene exhibited at least moderate resistance to panicle blast. These studies suggest that *Pigm* has a breeding potential in improving blast resistance of *geng* rice in Jiangsu province. However, due to the fact that the *Pigm* locus is from a landrace Gumei 4 and the adverse effect of *PigmR* on 1,000-seed weight reported by Deng et al. (2017), the breeding value of the *Pigm* locus in *geng* rice breeding project in Jiangsu province remains to be extensively evaluated.

In this study, we introduced *Pigm* gene into two high-yield and good-quality *geng* rice cultivars in Jiangsu by MAS and obtained a series of advanced backcrossing lines (ABLs) with *Pigm* gene and excellent performance in field. Furthermore, we comprehensively evaluated the resistance of ABLs to both seedling and panicle blast diseases using 184 *M. oryzae* isolates collected from multiple places in the lower region of the Yangtze River, especially in Jiangsu province. In addition, no tightly linked undesirable effects on important economical and agronomical traits were found in the introduction of *Pigm*. Particularly, one of the advanced lines has been authorized as a new variety in Jiangsu province, which showed excellent blast resistance as well as high yield and good quality. Together, our data infer that *Pigm* gene has a high breeding value in the *geng* rice breeding project toward broad-spectrum and durable blast resistance, and the resistant ABLs generated in this study will be of high value in breeding practice in future.

## Results

### Development of Advanced Backcross Lines With *Pigm* by Marker-Assisted Selection

**FIGURE 1:**
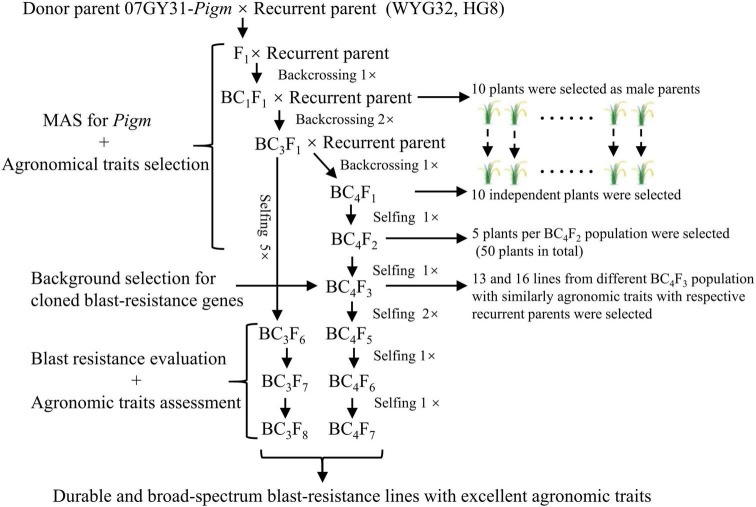
Marker-assisted selection (MAS) scheme for transferring *Pigm* and developing durable and broad-spectrum blast-resistant lines. WYG32, Wuyungeng 32; HG8, Huageng 8.

To determine the breeding potential of *Pigm* gene utilized in the geng rice breeding program in Jiangsu province, we introduced *Pigm* into two well-known *geng* rice cultivars WYG32 and HG8 by MAS, respectively (Figure 1). In BC_4_F_5_ generations, we obtained 13 and 16 ABLs, which contained homozygous *Pigm* gene and shared similar agronomic traits with the recurrent parents WYG32 and HG8, respectively. These lines were named WYG32-PG-1 to WYG32-PG-13 and HG8-PG-1 to HG8-PG-16 (Supplementary Table 1).

In order to exclude the influence of other rice blast *R* genes, we further detected the genotypes of these ABLs in other 13 known *R* gene loci (*Pia*, *Pib*, *Pid2*, *Pid3*, *Pik*, *Pikm*, *Pikh*, *Pit*, *Pita*, *Pi1*, *Pi5*, *Pi25*, and *Pb1*) using functional markers. We found that 11 of the 13 and 12 of the 16 ABLs carried same *R* gene combination as the recurrent parents WYG32 and HG8, respectively; and among them, five ABLs were selected from each background for subsequently resistant evaluation and agronomic trait assessment (Supplementary Table 1).

### Advanced Backcross Lines With *Pigm* Showed More Broad-Spectrum Resistance at the Seedling Stage to *M. oryzae* Strains Collected From the Lower Region of the Yangtze River

**FIGURE 2:**
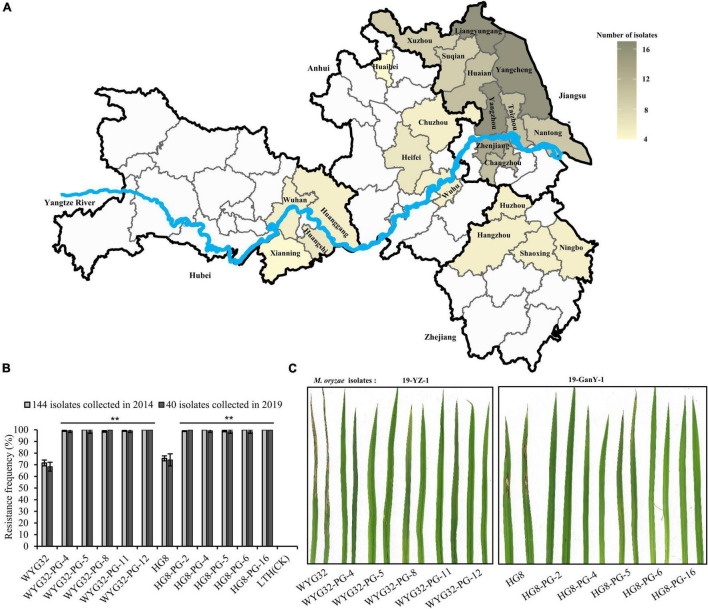
Blast resistance scores of ABLs and the recurrent parents at the seedling stage. **(A)** Distribution of 184 *M. oryzae* isolates used for seedling blast inoculation in the lower region of the Yangtze River. **(B)** Comparison of resistance frequencies among ABLs and the recurrent parents (WYG32 and HG8) against *M. oryzae* isolates. **(C)** Seedling blast disease reactions of ABLs against two *M. oryzae* isolates, 19-YZ-1 and 19-GanY-1. **P < 0.01 compared with the recurrent parent by Student’s *t*-test.

To evaluate the resistance effect of *Pigm* gene, a total of 144 *M. oryzae* isolates were used in inoculation assay. Among them, 84 strains were collected from infected fields in almost all prefectures of Jiangsu province, and the remaining isolates were from major rice growing provinces in the lower region of the Yangtze River (Figure 2A). The results showed that the susceptible variety LTH was susceptible to all 144 isolates, with a resistance frequency of 0 (Figure 2B), indicating that our inoculation assays were successful. The resistance frequencies of the recurrent parents WYG32 and HG8 were 71.53 and 75.46%, respectively, while the ABLs were found to be resistant to almost all isolates, with resistance frequencies ranging from 98.61 to 100% (Figure 2B). This indicates the *Pigm* has broad-spectrum resistance to *M. oryzae* isolates from the lower region of the Yangtze River.

To further determine the potential of *Pigm* in the current rice breeding program in Jiangsu province, we collected 85 isolates in 2019 from the locations that have been previously collected. Through functional markers of the 10 known avirulence gene loci (*Avr1-Co39*, *Avri-Pita*, *ACE1*, *Avr-Pib*, *PWL1*, *Avr-Pia*, *Avr-Pi9*, *Avr-Pizt*, *Avr-Pik*, and *Avr-Pii*), we found that these isolates had significant variation at the 10 known avirulence gene loci (Supplementary Table 2). Based on the phylogenetic results from genotyping data at these avirulence gene loci, we classified these 85 isolates into four groups (RA, RB, RC, and RD) (Supplementary Table 3). After combining the location information, we found that each of the four groups contained the isolates from all different ecological regions, implying that the genetic variation of these isolates was not caused by the ecological regions. Then, in each group, 10 isolates from different locations were selected and used in seedling blast inoculation assay. We found that all ABLs showed high resistance to these isolates with resistant spectra from 98.33 to 100%, which were significantly higher than those of the corresponding recurrent parent and the susceptible control LTH which showed a resistant spectrum of 0% (Figures 2B,C). Taken together, these data demonstrate that introducing *Pigm* into *geng* rice varieties in Jiangsu province is able to greatly broaden their resistance spectrum to blast disease, indicating a great potential of *Pigm* in the rice breeding program, especially in Jiangsu province.

### Advanced Backcross Lines With *Pigm* Exhibited High Resistance Against Panicle Blast

**FIGURE 3:**
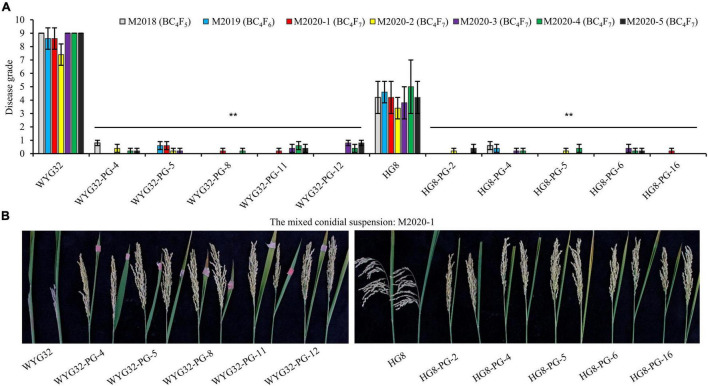
Panicle blast resistance scores of ABLs and the recurrent parents at the ripening stage. **(A)** Comparison of disease grades among ABLs and the recurrent parents (WYG32 and HG8) against the mixed conidial suspensions of *M. oryzae* isolates. **(B)** Panicle blast disease reactions of ABLs against the mixed conidial suspension M2020-1. **P < 0.01 compared with the recurrent parent by Student’s *t*-test.

To further assess the panicle blast resistance of these ABLs, a total of seven mixed conidial suspensions of the isolates collected from Jiangsu were used to inoculate ABLs at the booting stage. The mixed conidial suspensions of M2018, M2019, and M2020-1 were provided by Professor Yongfeng Liu, which were used in the test region of Jiangsu rice varieties for identifying the resistant level of each candidate rice variety in 2018, 2019, and 2020, respectively; four other mixed suspensions (M2020-2, M2020-3, M2020-4, and M2020-5) were prepared in the present study, each comprising six isolates that were selected from each of the four groups (RA, RB, RC, and RD) based on genotypes and location distribution (Figure 3 and Supplementary Table 3). In these seven trials, the disease grades of the control WYG32 were 9 in four trials (M2018, M2020-3, M2020-4, and M2020-5) and 7.4 ∼ 8.6 in the other three trials (M2019, M2020-1, and M2020-2), showing high susceptibility to panicle blast. By contrast, all five ABLs in this background reached a high resistance level to panicle blast, with the disease grade ranging from 0 to 0.8. For another background, the disease grades of HG8 were in the range of 3 ∼ 5 in all seven trials, exhibiting moderate resistance to panicle blast. However, all five ABLs under the HG8 background displayed high resistance to panicle blast, with the disease grade ranging from 0 to 0.6. Taken together, these results indicated that transferring *Pigm* into *geng* rice varieties in Jiangsu province could greatly improve rice resistance to panicle blast.

### Advanced Backcross Lines With *Pigm* Displayed High Blast Resistance in Blast Nursery Field

**FIGURE 4:**
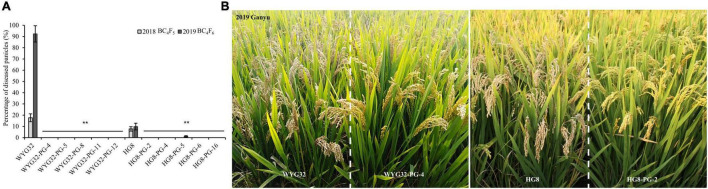
Resistance levels of ABLs to panicle blast in a blast nursery. **(A)** Comparison of diseased panicle rates among ABLs and the recurrent parents (WYG32 and HG8). **(B)** Panicle blast disease reactions of partial ABLs in the field in 2019. **P < 0.01 compared with the recurrent parent by Student’s *t*-test.

To evaluate the resistance effect of ABLs under natural conditions with high blast disease pressure, field assays were performed in Ganyu, Jiangsu province, in 2018–2019. As shown in Figure 4, the recurrent parent WYG32 showed moderate susceptibility in 2018 and high susceptibility in 2019 with diseased panicle rates of 17.7 and 92.3%, respectively, indicating the severity of this region on blast disease. However, as expected, the diseased panicle rates of all five ABLs under WYG32 background was 0% in both years, reaching a high resistant level. For another background, HG8 showed moderately resistant levels in 2 years, with diseased panicle rates of 7.8% in 2018 and 9.8% in 2019, whereas all the ABLs in this background showed high resistance in 2 years, except the line HG8-PG-5 which reached a resistant level of 1.1% in 2019. The results in the blast nursery further demonstrated that transferring *Pigm* into *geng* rice varieties in Jiangsu province could apparently enhance rice resistance to blast.

### No Apparently Adverse Effects Were Detected Among Advanced Backcross Lines With *Pigm* Compared With the Recurrent Parent

To check whether the introduction of *Pigm* could affect agronomic traits and grain quality, we measured these traits for ABLs and the recurrent parents. As listed in Table 1, we found that in total, most ABLs displayed similar traits as the corresponding recurrent parent. A few traits were found to have significant differences between some lines and the control, whereas these were not common among different ABLs. For instance, ABLs WYG32-PG-4, WYG32-PG-8, WYG32-PG-11, and HG8-PG-6 showed higher plant height than the corresponding recurrent parents, whereas the other ABLs displayed a plant height similar to that of the controls, indicating these changed traits were not closely linked with *Pigm*. Together, these data allowed us to infer that the introduction of *Pigm* has no tightly linked adverse effects on rice agronomical traits and grain quality, at least for the varieties tested in our study.

**TABLE 1:** Performance of basic agronomic and grain quality traits in ABLs and the recurrent parents.

Cultivars/lines	Plant height (cm)	Days to heading (d)	Panicle number	Panicle length (cm)	Grain number per panicle	1,000-seed weight (g)	Seed setting rate (%)	Chalkiness rate (%)	Amylose content (%)
WYG32	98.06 ± 2.23	98.0 ± 0.71	12.6 ± 0.55	17.94 ± 1.18	131.8 ± 5.63	26.85 ± 0.43	93.65 ± 1.01	15.08 ± 1.16	15.76 ± 0.59
WYG32-PG-4	102.48 ± 2.69**	99.2 ± 0.84*	13.2 ± 0.84	18.14 ± 1.23	132.6 ± 4.16	26.93 ± 0.44	93.10 ± 1.74	14.56 ± 0.81	15.28 ± 0.55
WYG32-PG-5	99.76 ± 2.10	97.4 ± 0.55	13.0 ± 0.71	18.64 ± 1.17	133.2 ± 4.15	26.96 ± 0.36	92.45 ± 1.55	15.30 ± 0.93	16.36 ± 0.87
WYG32-PG-8	103.94 ± 2.10**	97.2 ± 0.45	12.4 ± 1.14	17.86 ± 1.19	130.6 ± 3.97	26.79 ± 0.54	95.59 ± 2.16	15.64 ± 0.84	15.82 ± 0.98
WYG32-PG-11	104.74 ± 1.83**	100.2 ± 0.84**	13.6 ± 1.14	18.12 ± 0.95	131.2 ± 4.32	26.86 ± 0.39	95.49 ± 1.58	13.88 ± 0.56*	15.54 ± 0.55
WYG32-PG-12	99.42 ± 1.45	96.2 ± 0.84**	13.4 ± 0.89	17.62 ± 1.03	130.8 ± 3.70	26.76 ± 0.28	93.69 ± 2.10	14.28 ± 0.76	16.52 ± 1.00
HG8	100.80 ± 3.50	97.4 ± 1.14	14.2 ± 1.30	18.38 ± 0.90	136.0 ± 5.24	27.56 ± 0.37	92.41 ± 1.76	17.82 ± 0.76	19.18 ± 1.01
HG8-PG-2	101.28 ± 2.19	101.0 ± 0.71**	13.6 ± 0.55	18.86 ± 1.06	141.8 ± 4.82*	27.13 ± 0.33	93.40 ± 2.19	10.38 ± 1.36**	16.06 ± 0.72**
HG8-PG-4	102.34 ± 2.00	100.2 ± 0.84**	13.8 ± 0.84	19.04 ± 1.18	143.2 ± 4.09**	27.06 ± 0.39*	93.62 ± 1.52	11.22 ± 1.16**	16.40 ± 0.89**
HG8-PG-5	100.42 ± 1.83	95.4 ± 1.14**	14.4 ± 0.55	18.58 ± 1.42	138.2 ± 3.03	27.39 ± 0.48	95.51 ± 1.78*	16.94 ± 1.83	18.58 ± 0.90
HG8-PG-6	104.28 ± 2.43*	96.2 ± 1.10	13.6 ± 1.34	18.74 ± 1.15	140.8 ± 4.60	27.14 ± 0.29	92.04 ± 1.20	15.80 ± 2.13	17.38 ± 0.69**
HG8-PG-16	102.98 ± 1.51	97.8 ± 0.84	13.8 ± 0.84	18.48 ± 1.13	137.2 ± 3.83	27.51 ± 0.27	92.11 ± 3.19	18.92 ± 1.81	19.02 ± 0.92

*P < 0.05, **P < 0.01 compared with the corresponding respective recurrent parent by Student’s t-test.

The *Pigm* was reported previously to reduce grain weight but increase the seed setting ratio and ultimately balance grain yield and immunity (Deng et al., 2017). In the present study, however, we did not observe significant reduction on the grain weight of these ABLs. For these inconsistent results, we hypothesis that it could be due to various genes controlling grain weight distributed in these ABLs and Nipponbare, which were tested in a previous study. Therefore, we compared the genotypes of eight major genes controlling grain weight/shape (*TGW6*, *TGW3*, *GW2*, *GW5*, *GW8*, *qGL3*, *GS3*, and *qLGY3*) among ABLs Gumei 4 and Nipponbare by using functional markers (Gong et al., 2021). We found that all ABLs carried *TGW6*, *TGW3*, *GW2*, and *GS3*, whereas Gumei 4 and Nipponbare contained *GW2*, *GS3*, and *qGL3* (Supplementary Figure 1). Comparatively, *TGW6* and *TGW3* were specifically harbored in all ABLs, suggesting that one of them or both may offset the negative effect of *Pigm* on grain weight, which is of interest for further verification.

### A New Rice Variety Yangnonggeng 3091 Was Authorized With Strong Blast Resistance, High Grain Yield, and Good Quality

**FIGURE 5:**
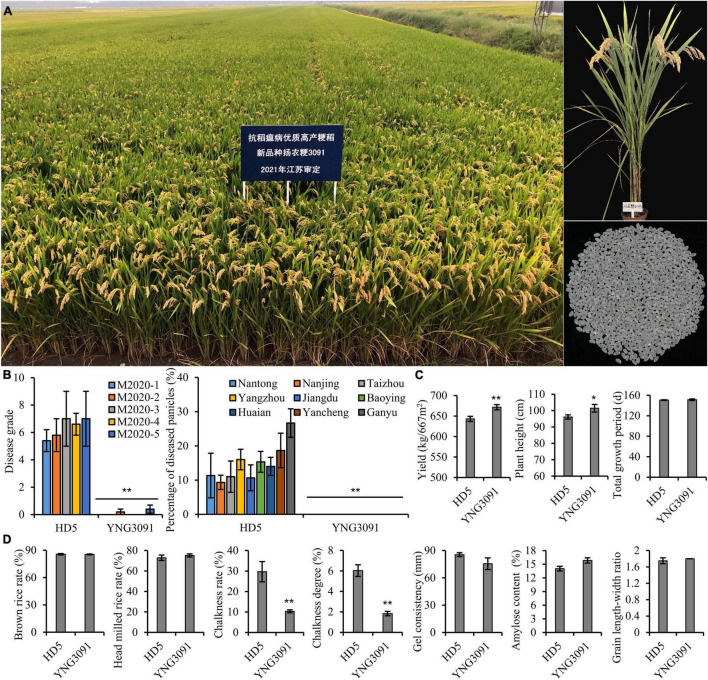
Breeding high blast-resistant rice variety Yangnonggeng 3091 (YNG 3091) with high yield and quality. **(A)** Field performance and milled rice grains of YNG3091. **(B–D)** Comparison of blast resistance levels **(B)**, important agronomic traits **(C)**, and grain quality traits **(D)** between YNG3091 and the control cultivar Huaidao 5 (HD5). *P < 0.05, **P < 0.01 compared with HD5 by Student’s *t*-test.

In addition to the MAS selection of *Pigm*, we also conducted agronomic trait selection and evaluation, which allowed us to obtain a new rice line in BC_3_F_6_ generation under HG8 background, designated as Yangnonggeng 3091(YNG3091, Figure 5A). In terms of panicle blast resistance, YNG3091 exhibited high resistance (disease grade, 0 ∼ 0.4) to all five mixed conidial suspensions (2020-1, 2020-2, 2020-3, 2020-4, and 2020-5), whereas the well-known control variety Huaidao 5 (HD5) in the rice region test of Jiangsu province showed moderately susceptible levels (disease grade, 5.4 ∼ 7) (Figure 5B). Moreover, YNG3091 recorded high resistance levels in natural blast disease nurseries in nine different cities of Jiangsu province (Figure 5B). For grain yield, we found that YNG3091 increased by 4.4% per 667 m^2^ compared with HD5 (Figure 5C). In addition, YNG3091 showed a slightly higher plant height than HD5 but had a similar total growth period. With respect to grain quality, we found that the chalkiness rate and chalkiness degree of YNG3091 were significantly lower than those of HD5, and the other traits were similar to those of HD5 (Figure 5D). Due to good performance on grain appearance, YNG3091 was evaluated as grade II of good quality rice according to the rice quality standard of China’s agricultural industry (MARAPRC, 2002). In 2021, YNG3091 was authorized as a new rice variety (Approval No.: Sushengdao 20210046) in Jiangsu province (Figure 5). This further confirmed that *Pigm* gene has great potential in developing new rice varieties with high blast resistance.

## Discussion

### *Pigm* Conferred Durable and Broad-Spectrum Resistance to Both Seedling and Panicle Blast

Deployment of broad-spectrum and durable resistance genes is of great importance to develop a broad-spectrum resistance variety. In this study, we developed several ABLs containing *Pigm* gene in the genetic background of two different *geng* rice varieties from Jiangsu province by MAS (Figure 1). Wang et al. (2020) found that in addition to the *Pigm* locus, 13 known *R* genes also distributed in *geng* rice varieties of Jiangsu province with various distribution degrees. A few *R* genes and *R* gene combinations showed apparently different contributions on rice blast resistance. Therefore, in order to reduce the effects from other *R* genes on the evaluation of *Pigm* resistance, we selected five ABLs in each background that showed same genotypes with the corresponding recurrent parent in the other 13 known *R* gene loci (Supplementary Table 1). After inoculation with 184 *M. oryzae* isolates in the seedling stage, we found that all ABLs showed significantly higher broad-spectrum resistance than the corresponding recurrent parent. These 184 isolates were from different locations and years in the lower region of the Yangtze River, especially in Jiangsu province, displayed a wide regional representative (Figure 2). It is expected that after years of planting different varieties, the major epidemic isolates in field might be changed. For further testing the resistance effect of *Pigm* on recent *M. oryzae* isolates, 40 of the 184 isolates were collected in 2019 from the locations that were previously collected in 2014, which showed an apparently genetic diversity based on the genotype analysis of known avirulence gene loci (Supplementary Tables 2,3). After combining the other studies using different isolates from different years and at least three different rice growing ecological regions (Tian et al., 2016; Dai et al., 2018; Zhou et al., 2021b), we infer that *Pigm* gene confers durable and broad-spectrum resistance to rice blast.

In terms of breeding practice, it is of great significance to regularly collect latest epidemic isolates to evaluate the resistance of rice lines to rice blast, especially panicle blast. The laboratory of Professor Yongfeng Liu (Plant Protection Institute of Jiangsu Academy of Agricultural Sciences) has been used to perform the evaluation experiment of blast resistance for the rice lines collected from the test region of new rice varieties in Jiangsu province. Therefore, they have to isolate a large number of *M. oryzae* isolates from all over the province each year, and through the pathogenicity test, six of the isolates that showed the strongest pathogenicity and genetic diversity were used for panicle inoculation next year (private communication with Prof. Liu). The mixed conidial suspensions M2018, M2019, and M2020-1 were from Liu’s laboratory, and we found that the ABLs showed a high resistance level to all these conidial suspensions in panicle blast inoculation assay. Similarly, in the inoculation assay using the other four mixed conidial suspensions, the ABLs reached high resistance levels (Figure 3 and Supplementary Tables 2,3). Meanwhile, the results of the natural evaluation in the blast nursery in Ganyu in 2 consecutive years (2018–2019) and nine different cities in 2020 showed a consistent trend with artificial inoculation (Figures 4, 5B). These results indicate that *Pigm* gene has strong effects in improving rice panicle blast resistance, at least for *geng* rice varieties in Jiangsu province. Collectively, although the fact that durable resistance of *Pigm* is still required to be monitored after a number of rice varieties with *Pigm* gene widely planted, the *Pigm* gene could be considered conferring durable resistance because it displayed very broad-spectrum resistance to both seedling and panicle blast.

### Effect of *Pigm* Gene on Reduction of Rice Grain Weight Can Be Probably Eliminated by Combination With Other Excellent Grain Weight-/Shape-Related Alleles

Linkage between *R* genes and deleterious alleles (linkage drag) is one of the major reasons limiting their utilization in breeding practice. Many broad-spectrum *R* genes, such as *Pi1*, *Pi9*, *Pi33*, *Pi40*, *Pi56*, and *Pigm*, are mainly distributed in local special germplasms but not modern cultivars, and their cumbersome chain of linkage drag might produce negative effects on agronomic traits (Xiao et al., 2020). The *Pigm* gene was reported to achieve yield balance by reducing grain weight but increasing the seed setting (Deng et al., 2017), while a decrease in grain weight is generally an undesirable trait in breeding practice. In the present study, we obtained five ABLs in each of the two recurrent parents. Notably, when compared with the corresponding recurrent parent, we found that all ABLs displayed almost the same 1,000-grain weight with the control, except for HG8-PG-4 which showed a significant lower 1,000-grain weight than HG8 (Table 1). Grain weight is typical of a quantitative trait controlled by multiple genes, and so far, at least 44 genes have been confirmed to be involved in the regulation of grain weight in rice (Li et al., 2018). Specially, wide interactions among different grain weight-related genes and complex affection of hormone signaling on grain weight have been found (Zuo and Li, 2014), which will undoubtedly affect the expression of a minor effect gene in complex genetic background. In the breeding process for developing these ABLs, the plants without good performance on agronomic traits, including grain size, were in fact not selected. Based on this, we further found that compared with Gumei 4 and Nipponbare, ABLs specifically carried *TGW6* and *TGW3* genes among eight major genes controlling the grain weight/shape (Supplementary Figure 1), suggesting that probably one of them or both may counteract the negative effect of *PigmR* on grain weight in ABLs, which is worthy of further investigation. In addition, due to the fact that the backcross and self-pollinated populations in each generation do not contain a large number of plants (Figure 1), we inferred that a weak reduction of *PigmR* on grain weight could be easily eliminated by agronomical trait selection or at least in the varieties’ background in this study.

Notably, a rice line YNG3091 from BC_3_F_6_ generation has been authorized as a new rice variety in Jiangsu province, which displays good resistance to both seedling blast and panicle blast, as well as excellent performance on both grain yield and quality (Figure 5). Most recently, a new *geng* variety Jinxiangyu 1 carrying *Pigm* was also developed *via* MAS in Jiangsu province, which also showed excellent blast resistance and high yield (Xiao et al., 2021). Together, these demonstrate that *Pigm* has a high breeding value in developing a blast-resistant variety and the reduction effect of *PigmR* on grain weight could be overcome by agronomical trait selection. In addition, this study provides several new rice lines including the variety YNG3091 carried *Pigm*, which can be directly used as new donors in the rice breeding project and ultimately speed up the development of blast resistant varieties in Jiangsu province and its surrounded region.

## Materials and Methods

### Plant Materials and Pathogens

The *geng* rice line 07GY31*-Pigm*, which carries *Pigm* from Gumei 4 and exhibits broad-spectrum resistance against rice blast (Wu et al., 2017), was used as the donor of *Pigm*, and two *geng* rice cultivars, Wuyungeng 32 (WYG32) and Huageng 8 (HG8), were authorized by Jiangsu province, which showed high yield and good grain quality, and selected as recurrent parents to develop blast-resistant materials/varieties with high breeding potential.

Totally, 144 and 85 *M. oryzae* isolates were collected and obtained from infected fields of 22 cities in the lower region of the Yangtze River (including Jiangsu, Anhui, Hubei, and Zhejiang provinces) in 2014 (Wu et al., 2017) and 13 different cities/counties in Jiangsu province in 2019, including Jingtan (JT), Wujin (WJ), Zhenjiang (ZJ), Yangzhou (YZ), Baoying (BY), Jiangdu (JD), Gaoyou (GY), Taizhou (TZ), Huaian (HA), Siyang (SiY), Ganyu (GanY), Sheyang (SheY), and Yancheng (YC). The isolation and cultivation of single-spore strains and inoculum preparation were carried out as described previously (Puri et al., 2009).

### Molecular Marker Assay

Genomic DNA was extracted from 1-month-old rice leaves and the mycelium of *M. oryzae* strains using the CTAB method (Zhang et al., 1992). The tightly linked molecular marker ZJ58.7 (2013–2018) and the functional marker GMR-3 (2019–2020) were selected to monitor *Pigm* (Yu et al., 2013; Wang et al., 2019). The functional markers used to detect 13 cloned blast R genes (*Pia*, *Pib*, *Pi-d2*, *Pi-d3*, *Pik*, *Pikm*, *Pik-h*, *Pit*, *Pi-ta*, *Pi1*, *Pi5*, *Pi25*, and *Pb1*) in rice plants and 10 isolated avirulence genes (*Avr1-Co39*, *Avri-Pita*, *ACE1*, *Avr-Pib*, *PWL1*, *Avr-Pia*, *Avr-Pi9*, *Avr-Pizt*, *Avr-Pik*, and *Avr-Pii*) in *M. oryzae* strains referred to the primer sequences described by Shen et al. (2019) and Wang et al. (2020), respectively. The reaction mixture (20 μL) for PCR amplification consisted of 2 μL DNA template (about 40 ng μL^–1^), 1 μL of each primer (10 μmol L^–1^), 2 μL MgCl_2_ (25 mmol L^–1^), 0.4 μL dNTP (10 mmol L^–1^), 2 μL 10 × PCR buffer, 0.2 μL Taq polymerase enzyme (5 U μL^–1^), and 11.4 μL ddH2O. PCR amplification was carried out using the following protocol: a pre-denaturation for 5 min at 95°C, followed by 35 cycles of 30 s at 94°C, 30 s at the annealing temperature, 1min at 72°C, and a final extension 72°C for 10 min. The PCR products were separated by electrophoresis on 4% agarose gels with ethidium bromide and photographed using a gel imaging system (Tanon 2500).

### Marker-Assisted Selection Procedure for Transferring *Pigm* and Developing New Lines

The MAS scheme for transferring *Pigm* into two genetic backgrounds is presented in Figure 1. *Pigm* was monitored in progenies from either backcrossed or self-pollinated plants using the molecular markers. The donor of *Pigm* (07GY31*-Pigm*) was crossed with each of the two recurrent parents (WYG32 and HG8) to obtain F_1_ plants in 2013. Then F_1_ plants of each cross were backcrossed with the corresponding recurrent parent till obtaining the 10 advanced independent BC_4_F_1_ plants. In 10 BC_4_F_2_ populations, five plants per BC_4_F_2_ population (50 plants in total) with a homozygous *Pigm* allele and good agronomic traits were screened for individually harvesting. In BC_4_F_3_ populations, a total of 13 and 16 lines with a similar agronomic phenotype with WYG32 and HG8 were selected, respectively. In each BC_4_F_3_ line, only one plant with excellent performance through visible selection was kept, and the same selection strategy was used in each BC_4_F_4_ line. Last, till BC_4_F_5_, the agronomic characters of each lines presented uniformity and were harvested together as a pure line. All BC_4_F_5_ homozygous lines were then examined for other 13 cloned blast *R* genes, *Pia*, *Pib*, *Pi-d2*, *Pi-d3*, *Pik*, *Pikm*, *Pik-h*, *Pit*, *Pi-ta*, *Pi1*, *Pi5*, *Pi25*, and *Pb1*, using functional markers. Finally, in each background, five BC_4_F_5_ ABLs with the same *R* gene combination on these 13 *R* gene loci as the recurrent parents were selected in 2018 for subsequent experiments (Supplementary Table 1). Meanwhile, in the BC_3_F_6_ generation, excellent lines carrying *Pigm* were screened to participate in the region test of rice new varieties in Jiangsu province.

### Evaluation for Seedling Blast Resistance by Artificial Inoculation

The evaluation of seedling blast resistance by artificial inoculation was performed according to the method described by Wu et al. (2015) with slight modifications in BC_4_F_6_ generation in 2019 and BC_4_F_7_ generation in 2020. Totally, 12 plants of each line/cultivar were grown on a plastic tray (60 cm × 30 cm × 4 cm) in the greenhouse maintained at 26–32°C. The highly susceptible cultivar Lijangxintuanheigu (LTH) was used as the susceptible control. There were three replicate trays for each blast isolate. For the experiment, 3-week-old seedlings were placed in a glass box (70 cm × 45 cm × 33 cm) and were sprayed with 30 ml of an isolate conidial suspension using a vacuum pump (4–7.8 kg/cm^2^). The inoculated seedlings were cultured in the glass box maintained at 25°C in the dark for 24 h and then were transferred to a transparent plastic box, covered with fresh-keeping film to maintain humidity, and grown under 12-h light at 28°C/12-h darkness at 25°C for 7 days. Disease reactions were recorded 7 days post-inoculation in accordance with standard procedures (Mackill and Bonman, 1992). Resistance levels of tested materials were represented by resistance frequency, which was calculated as follows: number of incompatible isolates/total number of isolates inoculated × 100% (Wu et al., 2015).

### Evaluation for Panicle Blast Resistance by Artificial Inoculation and Natural Induction

The artificial identification of panicle blast resistance in field was performed as described previously (Wu et al., 2017) using the mixed conidial suspension containing six representative *M. oryzae* isolates. A randomized complete block design was used with three biological replications. Each replication contained 10 rows and 10 plants per row, with a row spacing of 11.1 by 30 cm. At the booting stage, 10 panicles were injected with 1 mL of each mixed conidial suspension (5 × 10^4^ conidia/mL). At the ripening stage, the panicle blast disease scores were evaluated according to a ‘0–9’ rating method (MARAPRC, 2014). According to disease scores, each rice line was then classified into differently resistant levels: highly resistant (0 ≤ disease grade < 1), resistant (1.0 ≤ disease grade < 3), moderately resistant (3.0 ≤ disease grade < 5.0), moderately susceptible (5.0 ≤ disease grade < 7.0), susceptible (7.0 ≤ disease grade < 9.0), and highly susceptible (disease grade = 9.0).

All tested materials were also subjected to the determination of blast resistance in blast nursery in the field of Ganyu county, Lianyungang city, Jiangsu, with high blast disease pressure in 2018–2019. A randomized complete block design was used with three biological replications. Each replication contained 10 rows and 10 plants per row, with a row spacing of 11.1 by 30 cm. The resistance level in the blast nursery was evaluated based on the diseased-panicle rate, which was calculated as the number of diseased panicles divided by the total number of panicles and multiplied by 100 (Ministry of Agriculture and Rural Affairs of the People’s Republic of China, 2014). According to the diseased panicle rates, the tested materials with a diseased panicle rate of 0% were considered highly resistant, 0.1–5% were resistant, 5.1–10% were moderately resistant, 10.1–25% were moderately susceptible, 25.1–50% were susceptible, and 50.1–100% were highly susceptible.

### Examination of Importantly Agronomic and Grain Quality Traits

The ABLs were planted in the field at Yangzhou University, Jiangsu, China. The planting method was the same as described earlier (evaluation for panicle blast resistance). Overall, 10 plants between the second and ninth rows were randomly selected for measuring the importantly agronomic traits, which included plant height, days to heading, panicle number, panicle length, grain number per panicle, 1,000-seed weight, seed setting rate, and grain quality-associated traits, including chalkiness rate and amylose content according to the previous description (IRRI, 2002).

### Data Analysis

Data of blast disease severity and agronomic traits were sorted and plotted by Excel 2016. Significant differences between ABLs and the recurrent parents were analyzed using Student’s *t*-test in the SPSS 19.0 program. The cluster analysis of 271 *M. oryzae* isolates was performed by using SPSS 19.0.

## Data Availability Statement

The original contributions presented in this study are included in the article/Supplementary Material, further inquiries can be directed to the corresponding authors.

## Author Contributions

SZ, ZC, and ZF designed the research. ZF, MYL, ZX, PG, YW, KW, JZ, XW, JW, MCL, and KH performed the experiments and analyzed the data. ZF wrote the manuscript. SZ, ZC, AL, YD, and HC revised the manuscript. All authors read and approved the manuscript.

## Conflict of Interest

The authors declare that the research was conducted in the absence of any commercial or financial relationships that could be construed as a potential conflict of interest.

## Publisher’s Note

All claims expressed in this article are solely those of the authors and do not necessarily represent those of their affiliated organizations, or those of the publisher, the editors and the reviewers. Any product that may be evaluated in this article, or claim that may be made by its manufacturer, is not guaranteed or endorsed by the publisher.

## Abbreviations

*M. oryzae*: *Magnaporthe oryzae*
*R*: resistance
MAS: marker-assisted selection
WYG32: Wuyungeng 32
HG8: Huageng 8
ABLs: advanced backcross lines with *Pigm*
QRLs: quantitative resistance loci
YNG3091: Yangnonggeng 3091
HD5: Huaidao 5.
