# Supplementary material for: Development of Rice Variety With Durable and Broad-Spectrum Resistance to Blast Disease Through Marker-Assisted Introduction of Pigm Gene
Source: Front Plant Sci. 2022 Jul 22;13:937767. doi: 10.3389/fpls.2022.937767 (PMC9354813; doi:10.3389/fpls.2022.937767)
Supplement: Supplementary file 1 [file Data_Sheet_1.docx]

**Supplementary Table 1. The information of rice blast resistance genes carried in ABLs.**

| **Cultivars/lines** | **Generation** | **Blast resistance genes carried** |
| --- | --- | --- |
| WYG32 |  | Pikh\|Pib\|Pita |
| WYG32-PG-1 | BC4F5 | Pib\|Pita\|Pigm |
| WYG32-PG-2 | BC4F5 | Pikh\|Pib\|Pita\|Pigm |
| WYG32-PG-3 | BC4F5 | Pikh\|Pib\|Pita\|Pigm |
| WYG32-PG-4^&^ | BC4F5 | Pikh\|Pib\|Pita\|Pigm |
| WYG32-PG-5^&^ | BC4F5 | Pikh\|Pib\|Pita\|Pigm |
| WYG32-PG-6 | BC4F5 | Pikh\|Pib\|Pita\|Pigm |
| WYG32-PG-7 | BC4F5 | Pikh\|Pib\|Pita\|Pigm |
| WYG32-PG-8^&^ | BC4F5 | Pikh\|Pib\|Pita\|Pigm |
| WYG32-PG-9 | BC4F5 | Pikh\|Pib\|Pigm |
| WYG32-PG-10 | BC4F5 | Pikh\|Pib\|Pita\|Pigm |
| WYG32-PG-11^&^ | BC4F5 | Pikh\|Pib\|Pita\|Pigm |
| WYG32-PG-12^&^ | BC4F5 | Pikh\|Pib\|Pita\|Pigm |
| WYG32-PG-13 | BC4F5 | Pikh\|Pib\|Pita\|Pigm |
| HG8 |  | Pikh\|Pia\|Pib\|Pita |
| HG8-PG-1 | BC4F5 | Pikh\|Pia\|Pib\|Pita\|Pigm |
| HG8-PG-2^&^ | BC4F5 | Pikh\|Pia\|Pib\|Pita\|Pigm |
| HG8-PG-3 | BC4F5 | Pikh\|Pia\|Pib\|Pita\|Pigm |
| HG8-PG-4^&^ | BC4F5 | Pikh\|Pia\|Pib\|Pita\|Pigm |
| HG8-PG-5^&^ | BC4F5 | Pikh\|Pia\|Pib\|Pita\|Pigm |
| HG8-PG-6^&^ | BC4F5 | Pikh\|Pia\|Pib\|Pita\|Pigm |
| HG8-PG-7 | BC4F5 | Pikh\|Pia\|Pib\|Pita\|Pigm |
| HG8-PG-8 | BC4F5 | Pikh\|Pia\|Pib\|Pita\|Pigm |
| HG8-PG-9 | BC4F5 | Pia\|Pib\|Pita\|Pigm |
| HG8-PG-10 | BC4F5 | Pikh\|Pia\|Pib\|Pita\|Pigm |
| HG8-PG-11 | BC4F5 | Pikh\|Pib\|Pita\|Pigm |
| HG8-PG-12 | BC4F5 | Pikh\|Pia\|Pib\|Pita\|Pigm |
| HG8-PG-13 | BC4F5 | Pia\|Pib\|Pita\|Pigm |
| HG8-PG-14 | BC4F5 | Pikh\|Pia\|Pib\|Pita\|Pigm |
| HG8-PG-15 | BC4F5 | Pikh\|Pib\|Pita\|Pigm |
| HG8-PG-16^&^ | BC4F5 | Pikh\|Pia\|Pib\|Pita\|Pigm |

**^&^** Lines selected for blast resistance evaluation and agronomic traits investigation.

**Supplementary Table 2. The genotypes of 85 *M. oryzae* isolates collected from 13 different cities/counties in Jiangsu province in 2019 at 10 identified avirulence gene loci.**

| **Isolates** | ***Avr-Pia*** | ***Avr-Pizt*** | ***ACE1*** | ***Avr-Pik*** | ***Avr-Pii*** | ***Avr-Pita*** | ***AVR1-Co39*** | ***PWL1*** | ***Avr-Pib*** | ***Avr-Pi9*** |
| --- | --- | --- | --- | --- | --- | --- | --- | --- | --- | --- |
| 19-JT-1 | 0 | 1 | 0 | 1 | 1 | 0 | 0 | 0 | 1 | 1 |
| 19-JT-2 | 1 | 1 | 1 | 1 | 1 | 1 | 0 | 0 | 1 | 1 |
| 19-JT-3 | 0 | 1 | 1 | 1 | 1 | 1 | 0 | 0 | 1 | 1 |
| 19-JT-4 | 1 | 1 | 1 | 1 | 0 | 1 | 1 | 0 | 1 | 1 |
| 19-JT-5 | 0 | 1 | 1 | 1 | 1 | 0 | 0 | 0 | 1 | 1 |
| 19-JT-6 | 0 | 1 | 0 | 0 | 0 | 0 | 0 | 0 | 1 | 1 |
| 19-JT-7 | 0 | 1 | 1 | 0 | 0 | 0 | 0 | 0 | 1 | 1 |
| 19-WJ-1 | 0 | 1 | 1 | 0 | 0 | 0 | 0 | 0 | 1 | 1 |
| 19-WJ-2 | 1 | 1 | 1 | 0 | 0 | 0 | 0 | 0 | 1 | 1 |
| 19-WJ-3 | 1 | 1 | 1 | 0 | 1 | 0 | 1 | 0 | 1 | 1 |
| 19-WJ-4 | 1 | 1 | 1 | 0 | 1 | 0 | 1 | 0 | 1 | 1 |
| 19-WJ-5 | 1 | 1 | 1 | 0 | 1 | 1 | 1 | 0 | 1 | 1 |
| 19-WJ-6 | 0 | 1 | 1 | 0 | 0 | 0 | 0 | 0 | 1 | 1 |
| 19-ZJ-1 | 0 | 1 | 1 | 1 | 0 | 1 | 0 | 0 | 1 | 1 |
| 19-ZJ-2 | 0 | 1 | 1 | 1 | 0 | 0 | 0 | 0 | 1 | 1 |
| 19-ZJ-3 | 1 | 1 | 1 | 1 | 1 | 1 | 0 | 0 | 1 | 1 |
| 19-ZJ-4 | 0 | 1 | 1 | 1 | 1 | 0 | 0 | 0 | 1 | 1 |
| 19-ZJ-5 | 0 | 1 | 1 | 1 | 1 | 0 | 0 | 0 | 1 | 1 |
| 19-ZJ-6 | 0 | 1 | 1 | 1 | 1 | 1 | 0 | 0 | 1 | 1 |
| 19-ZJ-7 | 0 | 1 | 1 | 1 | 1 | 0 | 0 | 0 | 1 | 1 |
| 19-ZJ-8 | 0 | 1 | 1 | 1 | 1 | 0 | 0 | 0 | 1 | 1 |
| 19-ZJ-9 | 0 | 1 | 1 | 1 | 1 | 0 | 0 | 0 | 1 | 1 |
| 19-ZJ-10 | 0 | 1 | 0 | 0 | 0 | 0 | 0 | 0 | 1 | 1 |
| 19-ZJ-11 | 1 | 1 | 1 | 1 | 0 | 1 | 1 | 0 | 1 | 1 |
| 19-YZ-1 | 1 | 1 | 1 | 1 | 1 | 1 | 0 | 0 | 1 | 1 |
| 19-YZ-2 | 1 | 1 | 1 | 0 | 0 | 0 | 0 | 0 | 1 | 1 |
| 19-BY-1 | 0 | 1 | 0 | 0 | 0 | 0 | 0 | 0 | 1 | 1 |
| 19-JD-1 | 0 | 1 | 1 | 1 | 0 | 1 | 0 | 0 | 1 | 1 |
| 19-JD-2 | 0 | 1 | 1 | 1 | 0 | 0 | 0 | 0 | 1 | 1 |
| 19-JD-3 | 0 | 1 | 1 | 1 | 0 | 1 | 0 | 0 | 1 | 1 |
| 19-JD-4 | 1 | 1 | 1 | 0 | 1 | 1 | 1 | 0 | 1 | 0 |
| 19-GY-1 | 1 | 1 | 1 | 1 | 0 | 0 | 0 | 0 | 1 | 0 |
| 19-GY-2 | 0 | 1 | 1 | 0 | 0 | 0 | 0 | 0 | 1 | 1 |
| 19-GY-3 | 1 | 1 | 1 | 1 | 0 | 1 | 1 | 0 | 1 | 0 |
| 19-GY-4 | 0 | 1 | 1 | 1 | 0 | 0 | 0 | 0 | 1 | 0 |
| 19-GY-5 | 1 | 1 | 1 | 0 | 1 | 1 | 0 | 0 | 1 | 1 |
| 19-GY-6 | 1 | 1 | 1 | 0 | 1 | 1 | 1 | 0 | 1 | 1 |
| 19-GY-7 | 1 | 1 | 1 | 1 | 1 | 1 | 1 | 0 | 1 | 1 |
| 19-GY-8 | 0 | 1 | 1 | 0 | 0 | 0 | 0 | 0 | 1 | 1 |
| 19-TZ-1 | 0 | 1 | 1 | 1 | 1 | 0 | 0 | 0 | 1 | 1 |
| 19-TZ-2 | 0 | 1 | 1 | 1 | 1 | 0 | 0 | 0 | 1 | 1 |
| 19-TZ-3 | 0 | 1 | 1 | 1 | 1 | 1 | 0 | 0 | 1 | 1 |
| 19-TZ-4 | 0 | 1 | 1 | 1 | 1 | 0 | 0 | 0 | 1 | 1 |
| 19-TZ-5 | 0 | 1 | 1 | 1 | 1 | 0 | 0 | 0 | 1 | 1 |
| 19-TZ-6 | 0 | 1 | 1 | 1 | 1 | 1 | 0 | 0 | 1 | 1 |
| 19-TZ-7 | 0 | 1 | 1 | 1 | 1 | 1 | 0 | 0 | 1 | 1 |
| 19-TZ-8 | 0 | 1 | 1 | 1 | 1 | 0 | 0 | 0 | 1 | 1 |
| 19-TZ-9 | 1 | 1 | 0 | 1 | 1 | 1 | 1 | 0 | 1 | 1 |
| 19-TZ-10 | 0 | 1 | 1 | 1 | 1 | 1 | 1 | 0 | 1 | 1 |
| 19-HA-1 | 1 | 1 | 1 | 1 | 1 | 0 | 0 | 0 | 1 | 1 |
| 19-HA-2 | 1 | 1 | 1 | 1 | 0 | 0 | 0 | 0 | 1 | 1 |
| 19-HA-3 | 1 | 1 | 1 | 1 | 0 | 0 | 0 | 0 | 1 | 1 |
| 19-HA-4 | 1 | 1 | 1 | 0 | 0 | 1 | 1 | 0 | 1 | 1 |
| 19-HA-5 | 1 | 1 | 1 | 0 | 0 | 1 | 0 | 0 | 1 | 1 |
| 19-HA-6 | 1 | 1 | 1 | 0 | 0 | 0 | 0 | 0 | 1 | 1 |
| 19-HA-7 | 1 | 1 | 1 | 0 | 0 | 1 | 0 | 0 | 1 | 1 |
| 19-HA-8 | 0 | 0 | 0 | 0 | 0 | 0 | 0 | 0 | 0 | 1 |
| 19-HA-9 | 1 | 1 | 1 | 0 | 0 | 0 | 0 | 0 | 1 | 1 |
| 19-HA-10 | 0 | 1 | 1 | 0 | 1 | 0 | 0 | 0 | 1 | 1 |
| 19-HA-11 | 1 | 1 | 1 | 0 | 0 | 1 | 0 | 0 | 1 | 1 |
| 19-HA-12 | 1 | 1 | 1 | 1 | 0 | 0 | 1 | 0 | 1 | 1 |
| 19-SiY-1 | 1 | 1 | 1 | 0 | 0 | 0 | 0 | 0 | 1 | 1 |
| 19-SiY-2 | 1 | 1 | 1 | 0 | 0 | 0 | 0 | 0 | 1 | 1 |
| 19-SiY-3 | 1 | 1 | 1 | 0 | 0 | 0 | 0 | 0 | 1 | 1 |
| 19-SiY-4 | 0 | 1 | 1 | 1 | 0 | 0 | 0 | 0 | 1 | 1 |
| 19-SiY-5 | 1 | 1 | 1 | 0 | 0 | 0 | 0 | 0 | 1 | 1 |
| 19-SiY-6 | 1 | 1 | 1 | 0 | 0 | 0 | 0 | 0 | 1 | 1 |
| 19-SiY-7 | 1 | 1 | 1 | 0 | 0 | 0 | 0 | 0 | 1 | 1 |
| 19-SiY-8 | 1 | 1 | 1 | 0 | 0 | 0 | 0 | 0 | 1 | 1 |
| 19-SiY-9 | 0 | 1 | 1 | 0 | 0 | 0 | 0 | 0 | 1 | 1 |
| 19-SiY-10 | 0 | 1 | 1 | 1 | 0 | 0 | 0 | 0 | 1 | 1 |
| 19-GanY-1 | 0 | 1 | 1 | 0 | 0 | 0 | 0 | 0 | 1 | 1 |
| 19-GanY-2 | 0 | 1 | 1 | 1 | 0 | 1 | 0 | 0 | 1 | 1 |
| 19-GanY-3 | 0 | 1 | 1 | 1 | 0 | 0 | 0 | 0 | 1 | 1 |
| 19-GanY-4 | 1 | 1 | 1 | 0 | 1 | 0 | 0 | 0 | 1 | 1 |
| 19-GanY-5 | 0 | 1 | 1 | 1 | 0 | 0 | 0 | 0 | 1 | 1 |
| 19-GanY-6 | 0 | 1 | 1 | 0 | 1 | 1 | 0 | 0 | 0 | 1 |
| 19-GanY-7 | 0 | 1 | 0 | 0 | 1 | 0 | 0 | 0 | 0 | 1 |
| 19-GanY-8 | 0 | 1 | 1 | 0 | 0 | 0 | 0 | 0 | 1 | 1 |
| 19-GanY-9 | 0 | 1 | 0 | 0 | 0 | 0 | 0 | 0 | 1 | 1 |
| 19-SheY-1 | 1 | 1 | 1 | 1 | 0 | 0 | 0 | 0 | 1 | 1 |
| 19-SheY-2 | 1 | 1 | 1 | 1 | 0 | 1 | 0 | 0 | 1 | 1 |
| 19-YC-1 | 1 | 1 | 1 | 0 | 0 | 0 | 0 | 0 | 1 | 1 |
| 19-YC-2 | 0 | 1 | 1 | 0 | 0 | 1 | 0 | 0 | 1 | 1 |
| 19-YC-3 | 0 | 1 | 1 | 0 | 0 | 0 | 0 | 0 | 1 | 1 |

1 and 0 indicated that the gene was detected and not detected in this isolates, respectively. JT, Jingtan; WJ Wujin; ZJ, Zhenjiang; YZ, Yangzhou, BY, Baoying, JD, Jiangdu, GY, Gaoyou, TZ, Taizhou; HA, Huaian; SiY, Siyang; GanY, Ganyu; SheY, Sheyang; YC, Yancheng.

**Supplementary Table 3. The name and number of *M. oryzae* isolates in different groups.**

| **Group** | **Isolates name** | **Number** |
| --- | --- | --- |
| RA | 19-JT-6^*#^、19-JT-7、19-WJ-1、19-WJ-6、19-ZJ-10^*#^、19-BY-1^*#^、19-GY-2、19-GY-8、19-HA-8^*#^、19-SiY-9^*#^、19-GanY-1^#^、19-GanY-7^*^、19-GanY-8、19-GanY-9、19-YC-3^*^ | 15 |
| RB | 19-JT-3^*^、19-WJ-2、19-ZJ-1^*#^、19-ZJ-6、19-YZ-2、19-JD-1^*#^、19-JD-3、19-GY-5、19-TZ-3^*^、19-TZ-6、19-TZ-7、19-HA-5^*#^、19-HA-6、19-HA-7、19-HA-9、19-HA-11、19-SiY-1^*#^、19-SiY-2^*^、19-SiY-3、19-SiY-5、19-SiY-6、19-SiY-7、19-SiY-8、19-GanY-2^*#^、19-GanY-4^*^、19-GanY-6、19-YC-1^*#^、19-YC-2 | 28 |
| RC | 19-JT-2^*^、19-JT-4、19-WJ-3^*#^、19-WJ-4、19-WJ-5、19-ZJ-3^*#^、19-ZJ-11、19-YZ-1^*#^、19-JD-4^*^、19-GY-3^*#^、19-GY-6、19-GY-7、19-TZ-9^*#^、19-TZ-10^*^、19-HA-4^*#^、19-HA-12^*^ | 16 |
| RD | 19-JT-1^*#^、19-JT-5、19-ZJ-2^*^、19-ZJ-4、19-ZJ-5、19-ZJ-7、19-ZJ-8、19-ZJ-9、19-JD-2、19-GY-1^*^、19-GY-4、19-TZ-1^*#^、19-TZ-2、19-TZ-4、19-TZ-5、19-TZ-8、19-HA-1^*#^、19-HA-2^*^、19-HA-3、19-HA-10、19-SiY-4^*#^、19-SiY-10、19-SheY-1^*#^、19-SheY-2、19-GanY-3^*#^、19-GanY-5^*^ | 26 |

JT, Jingtan; WJ Wujin; ZJ, Zhenjiang; YZ, Yangzhou, BY, Baoying, JD, Jiangdu, GY, Gaoyou, TZ, Taizhou; HA, Huaian; SiY, Siyang; GanY, Ganyu; SheY, Sheyang; YC, Yancheng. ^*^Isolates selected for seedling blast resistance inoculation. ^#^Isolates selected for panicle blast resistance inoculation.


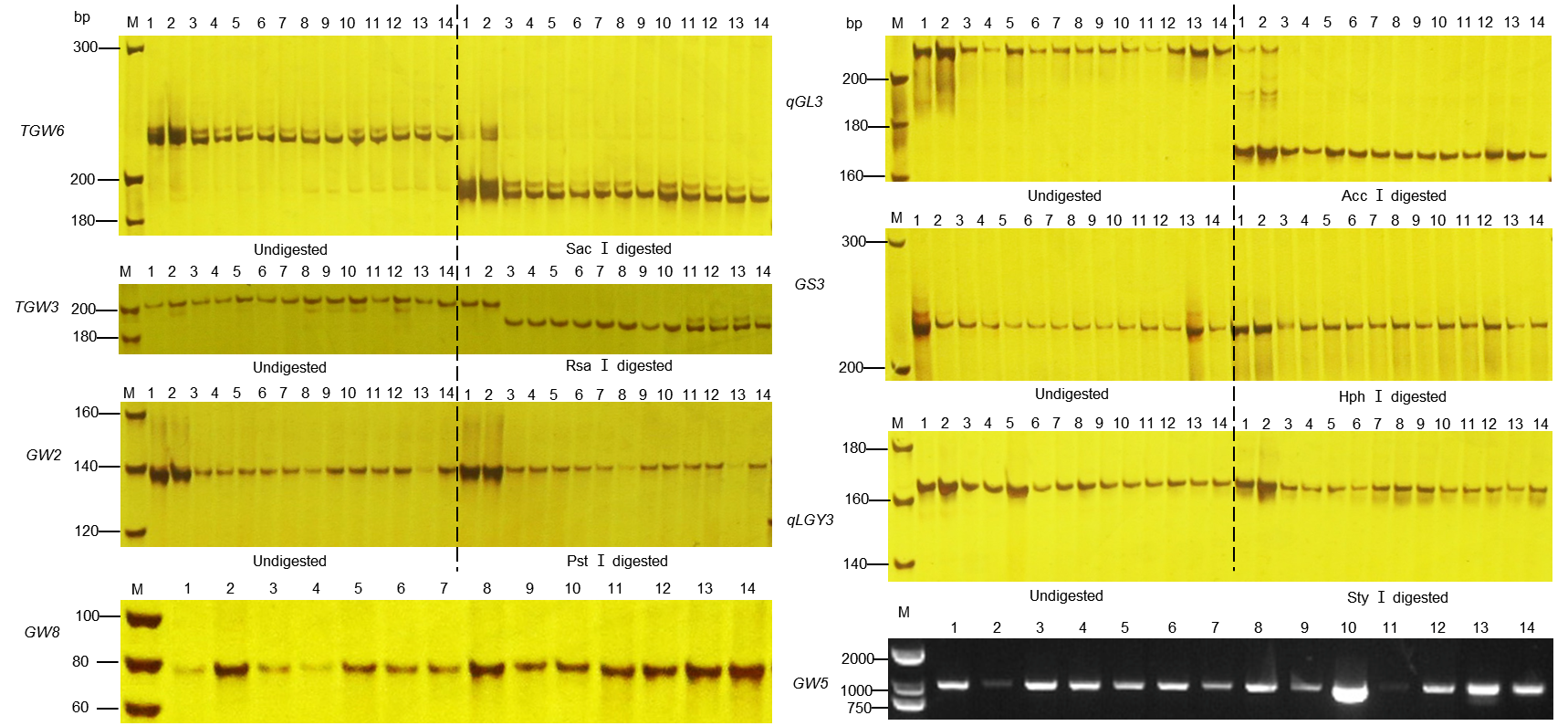


**Supplementary Figure 1.** Genotyping of eight grain weight/shape genes detected by dCAPS/Indel functional markers. 1-14: Nipponbare, Gumei 4, WYG32, WYG32-PG-4, WYG32-PG-5, WYG32-PG-8, WYG32-PG-11, WYG32-PG-12, HG8, HG8-PG-2, HG8-PG-4, HG8-PG-5, HG8-PG-6, and HG8-PG-16.
